# Genomic instability and genetic heterogeneity in aging: insights from clonal hematopoiesis (CHIP), monoclonal gammopathy (MGUS), and monoclonal B-cell lymphocytosis (MBL)

**DOI:** 10.1007/s11357-024-01374-y

**Published:** 2024-10-15

**Authors:** Attila Kallai, Zoltan Ungvari, Mónika Fekete, Andrea B. Maier, Gabor Mikala, Hajnalka Andrikovics, Andrea Lehoczki

**Affiliations:** 1 Healthy Aging Program, Institute of Preventive Medicine and Public Health, Semmelweis University, Budapest, Hungary; 2 Doctoral College, Health Sciences Program, Semmelweis University, Budapest, Hungary; 3 Vascular Cognitive Impairment, Neurodegeneration and Healthy Brain Aging Program, Department of Neurosurgery, University of Oklahoma Health Sciences Center, Oklahoma City, OK USA; 4 Stephenson Cancer Center, University of Oklahoma, Oklahoma City, OK USA; 5 Oklahoma Center for Geroscience and Healthy Brain Aging, University of Oklahoma Health Sciences Center, Oklahoma City, OK USA; 6 Department of Health Promotion Sciences, College of Public Health, University of Oklahoma Health Sciences Center, Oklahoma City, OK USA; 7 Doctoral College/Institute of Preventive Medicine and Public Health, International Training Program in Geroscience, Semmelweis University, Budapest, Hungary; 8 Department of Medicine and Aged Care, @AgeMelbourne, The Royal Melbourne Hospital, The University of Melbourne, Parkville, VIC Australia; 9 Department of Human Movement Sciences, @AgeAmsterdam, Faculty of Behavioural and Movement Sciences, Vrije Universiteit Amsterdam, Amsterdam Movement Sciences, Amsterdam, the Netherlands; 10 Centre for Healthy Longevity, @AgeSingapore, National University Health System, Singapore, Republic of Singapore; 11 Healthy Longevity Program, Yong Loo Lin School of Medicine, National University of Singapore, Singapore, @AgeSingapore, National University Health System, Singapore, Republic of Singapore; 12 Department of Hematology and Stem Cell Transplantation, Central Hospital of Southern Pest, National Institute for Hematology and Infectious Diseases, Budapest, Hungary; 13 Laboratory of Molecular Genetics, Central Hospital of Southern Pest, National Institute for Hematology and Infectious Diseases, Budapest, Hungary

**Keywords:** Biological age, Biomarker, Aging, Clock, Mutation

## Abstract

Aging is a multifaceted process characterized by a gradual decline in physiological function and increased susceptibility to a range of chronic diseases. Among the molecular and cellular mechanisms driving aging, genomic instability is a fundamental hallmark, contributing to increased mutation load and genetic heterogeneity within cellular populations. This review explores the role of genomic instability and genetic heterogeneity in aging in the hematopoietic system, with a particular focus on clonal hematopoiesis of indeterminate potential (CHIP), monoclonal gammopathy of undetermined significance (MGUS), and monoclonal B-cell lymphocytosis (MBL) as biomarkers. CHIP involves the clonal expansion of hematopoietic stem cells with somatic mutations. In contrast, MGUS is characterized by the presence of clonal plasma cells producing monoclonal immunoglobulins, while MBL is characterized by clonal proliferation of B cells. These conditions are prevalent in the aging population and serve as measurable indicators of underlying genomic instability. Studying these entities offers valuable insights into the mechanisms by which somatic mutations accumulate and drive clonal evolution in the hematopoietic system, providing a deeper understanding of how aging impacts cellular and tissue homeostasis. In summary, the hematopoietic system serves as a powerful model for investigating the interplay between genomic instability and aging. Incorporating age-related hematological conditions into aging research, alongside other biomarkers such as epigenetic clocks, can enhance the precision and predictive power of biological age assessments. These biomarkers provide a comprehensive view of the aging process, facilitating the early detection of age-related diseases and hopefully enabling personalized healthcare strategies.

## Introduction

Aging is a complex and multifaceted process characterized by a gradual decline in physiological function and increased vulnerability to diseases, ranging from cardiovascular diseases, neurodegenerative diseases, and musculoskeletal diseases to cancers. Over the past two decades, significant advances in research have elucidated various molecular and cellular mechanisms that drive aging, collectively known as the hallmarks of aging [1–4]. These hallmarks provide a comprehensive framework for understanding the biological underpinnings of aging and include genomic instability, telomere attrition, epigenetic alterations, impaired cellular resilience and loss of proteostasis, deregulated nutrient sensing, mitochondrial dysfunction and oxidative stress, cellular senescence, stem cell exhaustion, and altered intercellular communication [4–6].

Among these hallmarks, genomic instability stands out as a fundamental mechanism contributing to aging [6–15]. Genomic instability encompasses a range of genetic alterations, including mutations, chromosomal aberrations, and DNA damage, which accumulate over time [10, 16–22]. This DNA damage accumulation results in increased mutation load and genetic heterogeneity (“somatic mosaicism” [23]) within dividing cellular populations, ultimately impairing tissue function and organismal health [24–39]. The accumulation of mutations with age varies significantly between different organs and cell types [25, 34–36, 40–46]. Somatic mosaicism predominantly affects tissues with high rates of cell turnover (e.g., skin, liver, and hematopoietic cells) [23]. The role of age-related genomic instability is especially important in stem cells [44]. Stem cells are responsible for the maintenance and regeneration of tissues throughout the life of an organism. As stem cells divide, they pass on accumulated genetic damage to their progeny, leading to clonal expansion of mutated cells. This can disrupt tissue homeostasis and contribute to the functional decline seen in aging tissues.

The hallmarks of aging are interconnected: age-related oxidative stress is a critical driver of DNA damage, while impaired DNA repair is a key component of the age-related decline in cellular resilience [4]. Many of these age-related changes are regulated at the level of the epigenome, where modifications in DNA methylation and histone modification patterns influence gene expression and cellular function. Cellular senescence, a state of permanent cell cycle arrest in response to stress, is closely linked to DNA damage. Increased cellular senescence with aging is causally associated with heightened DNA damage, contributing to the overall decline in tissue function and increased susceptibility to age-related diseases [18, 28, 29]. These interconnected processes underscore the complexity of aging and highlight the central role of genomic instability.

Understanding genomic heterogeneity in aging is crucial because it provides insights into the dynamic and adaptive nature of cellular populations in response to genomic stress. As cells acquire mutations, they diversify, leading to clonal expansions and shifts in tissue composition [47–56]. This heterogeneity not only reflects the underlying genomic instability but also influences the aging process and the development of age-related diseases.

Much of our understanding of genomic heterogeneity in aging stems from cross-sectional studies, which provide snapshots of genetic diversity at specific points in time across different age groups. However, studying genomic instability longitudinally, especially in humans, presents significant challenges. One primary obstacle is the invasive nature of obtaining repeated biopsies from the same individual over extended periods. While cross-sectional studies offer valuable insights, longitudinal studies are crucial for understanding the dynamics of genomic instability and its impact on aging at the individual level.

A particularly valuable organ system for studying the interplay between genomic instability and genetic heterogeneity in humans is the bone marrow [46, 57–59]. The bone marrow is a rich source of hematopoietic stem cells (HSCs) and progenitor cells, making it an ideal model for investigating how genetic changes accumulate and propagate within a highly dynamic cellular environment [23, 46]. For example, using single-cell whole-genome sequencing, researchers found that somatic mutations in B lymphocytes increase from less than 500 per cell in newborns to over 3000 per cell in centenarians, with mutational hotspots and B cell-specific mutation signatures [46]. In recent years, conditions such as clonal hematopoiesis of indeterminate potential (CHIP) [23], monoclonal gammopathy of undetermined significance (MGUS), and monoclonal B-cell lymphocytosis (MBL) [60, 61] have emerged as measurable consequences of genomic instability in the bone marrow. These clonal hematopoietic entities are characterized by the presence of nonmalignant clonal cell populations that arise from somatic mutations and mosaic chromosomal alterations, and they are increasingly recognized as biomarkers of aging.

In this review, we will explore the mechanisms by which genomic instability contributes to aging, with a focus on the hematopoietic system. We will highlight the significance of CHIP, MGUS, and MBL as biomarkers of aging and discuss their potential to complement other aging biomarkers, such as epigenetic clocks. By understanding the role of genetic heterogeneity and genomic instability in human aging, we can gain valuable insights into the aging process and identify new avenues for therapeutic intervention.

## Genomic instability and aging

Genomic instability refers to the increased frequency of mutations within the genome, encompassing a broad spectrum of genetic alterations such as point mutations, insertions and deletions, chromosomal rearrangements, and aneuploidy. These alterations arise from intrinsic factors, including DNA replication errors and reactive oxygen species (ROS), as well as extrinsic factors, such as environmental toxins and radiation. The cellular machinery responsible for maintaining genomic integrity, including DNA repair pathways, the cell cycle checkpoint, and apoptosis, becomes less efficient with age. This decline in genomic maintenance allows genetic damage to accumulate, leading to increased genomic instability.

### Role of mutation load in genetic heterogeneity

As organisms age, the accumulation of genetic mutations contributes to an increasing mutation load within cells [24–39]. This heightened mutation burden results in significant genetic heterogeneity among cellular populations [24–39]. In tissues with high cell turnover, such as the hematopoietic system, this heterogeneity is particularly pronounced. Each stem cell acquiring unique mutations leads to a mosaic of genetically distinct clones within the tissue. This clonal diversity can drive various cellular behaviors, with some clones potentially gaining a proliferative advantage or resistance to apoptosis. Consequently, this genetic heterogeneity is a direct outcome of the continuous accumulation of mutations, reflecting the underlying genomic instability.

### Impact of genomic instability on tissue function and aging

Genomic instability and the resultant genetic heterogeneity have profound implications for tissue function and overall aging [23, 41, 42, 62, 63]. As genetically diverse clones expand, they can disrupt the normal architecture and function of tissues. For instance, in the bone marrow, clonal expansions associated with conditions like CHIP and MGUS can impair hematopoiesis, leading to reduced production of healthy blood cells and increased risk of hematological malignancies. Additionally, these conditions are associated with an increased incidence of cardiovascular aging phenotypes, including a higher risk of thrombotic events and cardiovascular diseases [49, 50, 56, 64–70]. Additionally, genomic instability can induce cellular senescence, a state of irreversible growth arrest, contributing to the depletion of stem cell pools and impairing tissue regeneration [29]. The cumulative effect of these processes accelerates the functional decline of tissues, manifesting as the phenotypic hallmarks of aging and increasing susceptibility to age-related diseases. Understanding the impact of genomic instability is crucial for developing strategies to mitigate its effects and promote healthy aging.

## Hematopoietic system: a model for studying aging processes

The hematopoietic system is a complex network responsible for the production and regulation of blood cells. It encompasses the bone marrow, where hematopoietic stem cells (HSCs) reside and differentiate into various blood cell lineages, including red blood cells, white blood cells, and platelets. This system also includes peripheral blood, lymphoid tissues, and organs such as the spleen and thymus, which are essential for immune function and blood cell maturation. The hematopoietic system maintains homeostasis through a finely tuned balance of cell proliferation, differentiation, and apoptosis, ensuring a constant supply of functional blood cells throughout an individual’s life.

### Relevance of the hematopoietic system in aging research

As individuals age, the functionality of the hematopoietic system declines, leading to reduced regenerative capacity, impaired immune responses, and increased susceptibility to diseases, including infections, anemia, and hematological cancers [71–74]. Age-related changes in the hematopoietic system include the exhaustion of HSCs, shifts in cell lineage potential, loss of reconstitution potential, and alterations in the bone marrow microenvironment. These changes provide a window into the broader impacts of aging on tissue function and organismal health, making the hematopoietic system an excellent model for studying the mechanisms of aging.

One significant application of studying the hematopoietic system in aging research is the use of blood cell analysis to determine biological age [75]. Biological age, which may differ from chronological age, reflects an individual’s physiological state and susceptibility to age-related diseases [75–78]. Standard laboratory parameters derived from blood cell analysis, such as white blood cell counts and lymphocyte-to-monocyte ratios, have been incorporated into various “biological clocks” [75, 78]. These clocks are computational models that estimate biological age by integrating multiple hematological parameters with other laboratory parameters and/or physiological measurements [78]. Such measures provide a comprehensive assessment of an individual’s health and biological aging, offering the potential for early detection of age-related decline and guiding interventions to promote healthy aging.

### Benefits of using the hematopoietic system to study genomic instability and genetic heterogeneity

The hematopoietic system offers numerous advantages for studying genomic instability and genetic heterogeneity [23]. Its high cellular turnover and the presence of long-lived HSCs make it highly susceptible to the accumulation of genetic mutations over time. These mutations contribute to clonal hematopoiesis, where specific cell clones expand due to their growth advantage, resulting in a diverse genetic landscape within the hematopoietic system.

One notable condition related to genomic instability in the hematopoietic system is CHIP. CHIP is characterized by the presence of clonal blood cell populations derived from HSCs that harbor somatic mutations or mosaic chromosomal alterations [49–51, 70, 79–81]. These clonal populations can impact hematopoiesis and are associated with an increased risk of hematological malignancies and cardiovascular disease, making CHIP a valuable biomarker for aging research [49–51, 70, 79–81].

CHIP, as defined, is usually not associated with detectable peripheral blood count abnormalities. The progression of clonal myeloid cells may result in clonal cytopenias similar to those observed in myelodysplasia (MDS) or, rarely, polycythemia/thrombocytosis similar to those observed in myeloproliferative neoplasm (MPN). On the other hand, the progression of clonal lymphoid cells may result in monoclonal B-cell lymphocytosis (MBL) or, in rare instances, T-cell clones of uncertain significance (TCUS) [82]. Additionally, the proliferation of clonal B cells that mature to produce abnormal monoclonal immunoglobulins consequently leads to monoclonal gammopathy of unknown significance (MGUS) [83–87]. MGUS is considered a precursor to multiple myeloma, other plasma cell dyscrasias, and more rarely to other lymphoproliferative diseases and is more prevalent with advancing age [83–85]. Studying MGUS provides insights into how genomic instability and clonal expansions in B cells contribute to aging and disease.

The effects of receptor-diverse and homogeneous clonal hematopoiesis differ in their impact on immune function and disease risk. Receptor-diverse clones, which arise due to antigenic stimulation, tend to maintain a higher level of immune competence, preserving a range of T-cell or B-cell receptors. In contrast, homogeneous clonal hematopoiesis, where a single dominant clone expands, can reduce immune diversity and increase susceptibility to infections or immune dysregulation, as seen in conditions such as MGUS and MBL. This diversity or lack thereof may also influence the risk of progression to hematologic malignancies, with homogeneous clones potentially harboring a higher risk due to the lack of immune regulation and increased vulnerability to additional mutations.

Advances in single-cell sequencing and other high-throughput techniques have enhanced our ability to analyze the genetic and functional diversity of cells within the hematopoietic system [40, 46, 73]. These technologies allow for detailed mapping of mutation patterns, clonal dynamics, and cellular behaviors, providing a comprehensive understanding of how genomic instability drives genetic heterogeneity and impacts aging [88–91].

## Clonal hematopoiesis of indeterminate potential (CHIP)

### Definition and prevalence of CHIP

CHIP is characterized by the presence of an expanded clone of hematopoietic cells that harbor somatic mutations in genes commonly associated with hematologic malignancies in the absence of overt blood cancer or other hematologic abnormalities [23, 49, 50, 67, 70, 80, 81]. The present definition established the variant allele fraction (VAF) of ≥ 2% of somatic alterations of hematologic malignancy-associated genes, because the clinical consequence of variants below this threshold is less detectable [61]. CHIP is identified through genetic sequencing of blood cells, revealing specific mutations that confer a growth or survival advantage to particular cell clones [53, 54, 56, 92]. The prevalence of CHIP increases with age, affecting approximately 10–20% of individuals over the age of 70 [66, 80, 93]. Although it is asymptomatic and often detected incidentally, CHIP is a significant marker of clonal expansion within the hematopoietic system [23, 53, 54, 63, 93, 94].

### Mechanisms leading to CHIP and its association with aging

The development of CHIP is driven by somatic mutations and mosaic chromosomal alterations affecting genes involved in hematopoietic cell regulation [23, 48, 49, 51, 53, 54, 56, 63, 64, 67, 80, 92, 95, 96]. These mutations occur in HSCs and confer a selective growth advantage preferentially to myeloid or lymphoid lineages, allowing the mutant clones to expand over time. Both myeloid and lymphoid driver gene mutations and mosaic chromosomal abnormalities can be detected, distinguishing between myeloid and lymphoid CHIP (M-CHIP and L-CHIP). Recent studies indicate that M-CHIP is more prevalent than L-CHIP [97]. Age-related declines in DNA repair mechanisms facilitate the accumulation of these mutations, increased oxidative stress, and the overall aging of the hematopoietic stem cell pool. As a result, both M- and L-CHIP become more prevalent with advancing age, reflecting the broader phenomenon of genomic instability associated with the aging process. In M-CHIP, somatic mutations in three genes *DNMT3A*, *TET2*, and *ASXL1* (DTA) were implicated in approximately 80% of cases, all involved in epigenetic regulation. On the contrary, L-CHIP variants were more evenly spread across a larger number of genes [23, 97]. Several genes connected to L-CHIP are involved in DNA damage response, chromatin modifications, and maintaining genomic integrity, such as *ATM*, *KMT2D*, *KMT2C*, and *SPEN* [97].

### Clinical implications of CHIP in the context of aging and disease

Although CHIP is believed to be asymptomatic, its presence has significant clinical implications [23, 49, 51, 52, 56, 67, 70, 93–95]. CHIP is a premalignant condition: individuals with CHIP have an increased risk of developing hematologic malignancies due to the potential for further genetic mutations that drive malignant transformation [51, 92, 98]. Myeloid and lymphoid CHIP variants clearly differentiate the type of malignancies: M-CHIP increases the likelihood of myeloid malignancies, while L-CHIP may lead to a higher incidence of lymphoid malignancies [97]. Additionally, preclinical and clinical studies indicate that the M-CHIP clonal events contribute to abnormal inflammatory and metabolic changes [23]. In humans, M-CHIP is associated with an elevated risk of cardiovascular disease, including atherosclerotic vascular disease leading to myocardial infarction [48–50, 56, 64, 67, 70, 79, 81] and stroke [66, 96], consequently adversely influencing the overall survival. The cardiovascular complications associated with M-CHIP may be explained by the loss of function related to the three main driver genes. However, the mechanisms underlying this association are not fully understood but may involve the pro-inflammatory effects of clonal hematopoietic cells, their migration to atherosclerotic lesions, and their contribution to both local and systemic inflammation [48–50, 56, 64, 67, 70, 79, 81]. On the contrary, L-CHIP is not associated with cardiovascular complications and increased mortality [97].

Beyond its role as a precursor to malignancy and cardiovascular disease, CHIP serves as a valuable biomarker for aging and age-related health risks [52]. Its detection can inform risk stratification and guide monitoring strategies for individuals at higher risk of adverse outcomes [52, 65, 99–103]. An important large-scale study by Nachun et al. demonstrated that CHIP is strongly associated with epigenetic age acceleration in multiple clocks [52]. Similar conclusions were reached by other studies as well [103]. CHIP carriers with mutations in multiple CHIP genes exhibit the most significant epigenetic age acceleration, which also associates with decreased telomere length [52]. The association of CHIP and epigenetic age acceleration identified a population at high risk of all-cause mortality (HR, 2.9) and coronary heart disease (HR, 3.24) [52]. Thus, the assessment of CHIP as a biomarker of age-related genomic instability offers potential avenues for early intervention and prevention of age-related diseases in high-risk populations.

## Clonal cytopenia of unknown significance (CCUS)

### Definition and prevalence of CCUS

Although the presence of CHIP mutations usually does not affect red cell distribution width (RDW), clonal cytopenia of undetermined significance (CCUS) [104–106] is characterized by the presence of M-CHIP along with one or more persistent cytopenias that cannot be explained by other hematologic or non-hematologic conditions and do not fulfill the diagnostic criteria for specific myeloid neoplasms. The definitions of cytopenias for CCUS, myelodysplastic syndromes (MDS), and MDS/myeloproliferative neoplasms (MDS/MPN) are harmonized and include the following: hemoglobin levels below 12–13 g/dL in females and males for anemia, an absolute neutrophil count below 1.8 × 10^9^/L for leukopenia, and platelet counts below 150 × 10^9^/L for thrombocytopenia. Anemia is the most prevalent form of cytopenia in older adults and serves as a key clinical and phenotypic marker of hematopoietic aging.

### Mechanisms leading to CCUS and its association with aging: clinical applications

Anemia in the elderly is most commonly attributed to nutrient deficiencies and chronic inflammation (ACI) [107, 108]. Despite extensive diagnostic resources, the cause of anemia remains unidentified in around one-third of the cases, regarded as unexplained anemia of the elderly (UAE) [109]. A large case–control study investigating the background of anemia revealed a higher prevalence of clonal hematopoiesis (M-CHIP) in cases with ACI and UAE compared to cases with nutrient deficiencies [110]. Although the occurrence of commonly detected DTA mutations did not differ between anemic and control cases, the anemia group exhibited a higher frequency of other mutations, including TP53 and SF3B1 [110]. Even a mild form of anemia in the elderly is correlated with significant morbidity and mortality [110].

## Monoclonal B-cell lymphocytosis (MBL)

### Definition, classification, and prevalence of MBL

MBL is characterized by the presence of a small monoclonal chronic lymphocytic leukemia/small lymphocytic lymphoma (CLL/SLL) phenotype B-cell clone in peripheral blood without diagnostic signs of B-lymphoproliferative disorder [60, 111–113]. In populations above the age of 40, the prevalence is estimated to be around 5–15% [114], and it rises to approximately 35% in individuals aged 70–79 years [111–113, 115].

MBL cases are categorized into low-count MBL (< 0.05 × 10^9^/L) and high-count MBL (0.05–0.5 × 10^9^/L) based on the number of clonal lymphocytes, with high-count MBL comprising 5–15% of all MBL cases [116]. This subset has the potential to progress to chronic lymphocytic leukemia/small lymphocytic lymphoma (CLL/SLL) [116]. Immunophenotyping further divides MBL into three subtypes: CLL-type MBL (representing 75–85% of cases), atypical CLL-type MBL, and non-CLL-type MBL [117]. CLL-type MBL is the most common and shares many features with CLL, while the atypical and non-CLL types are less common and exhibit different immunophenotypic characteristics [117]. This classification aids in understanding the clinical significance and potential progression of MBL to more serious conditions.

### Mechanisms leading to MBL

MBL emerges as the immune system is continuously stimulated throughout our lives, with genotypic changes occurring during each cell division. Certain genotypic alterations drive the expansion of B cells, leading to the propagation and formation of clones. These clones can retain their evolutionary advantages, continuing to expand even if the initial trigger stimuli are no longer present. Whole-genome sequencing of both low- and high-count MBL, as well as ultra-stable CLL (patients who do not show progression for over 10 years from initial diagnosis), revealed indistinguishable genomic profiles with low genomic complexity at both chromosomal and gene levels. The presence of somatic mutations in these cases indicated similar alterations, which likely occur before disease onset, probably at the hematopoietic stem cell level [118]. This suggests that MBL shares a common genetic background with CLL, including clinically relevant copy number alterations on a chromosomal level such as del13q, trisomy 12, del11q, and del17p [119], as well as gene mutations in TP53, NOTCH1, and SF3B1.

### Clinical implications of MBL in the context of aging and disease

MBL is an asymptomatic, premalignant stage of CLL with approximately a 1% annual risk of progression to CLL [120]. While individuals with low-count MBL have a 4.3 times higher risk of developing lymphoid malignancies compared to the general population, those with high-count MBL face a dramatically increased risk, estimated to be 74 times higher [116].

Monoclonality in MBL leads to a degree of immune impairment, increasing susceptibility to infections [115, 121]. This immune dysfunction is due to the clonal nature of the B cells, which affects the overall diversity and function of the immune system. Some studies have demonstrated decreased overall survival even in individuals with low-count MBL [122], although the literature is not entirely conclusive on this point, particularly regarding survival outcomes in low-count MBL cases [116].

## Monoclonal gammopathy of undetermined significance (MGUS)

Monoclonal gammopathy of undetermined significance (MGUS) is a condition characterized by the presence of an abnormal monoclonal protein (M protein) in the blood, produced by a clone of plasma cells [83, 87, 90]. Unlike multiple myeloma and other plasma cell dyscrasias, MGUS typically does not cause significant symptoms, organ damage, or a high level of M protein. MGUS is most commonly detected through blood tests conducted for unrelated reasons. It is a common condition, particularly among older adults, with a prevalence of over 5% in individuals 70 years of age or older and increasing to over 7.5% in those 85 years of age or older [83]. In the recent iStopMM study, the Icelandic population above the age of 40 years was systematically screened for the presence of MGUS, and 3.9% was found positive (5% in the > 50 years old population) [123, 124].

### Mechanisms leading to MGUS and its association with aging

MGUS is a premalignant condition that precedes multiple myeloma and carries an overall 1% annual risk of progression [125, 126]. The development of MGUS is believed to result from genetic and environmental factors that contribute to the clonal expansion of plasma cells producing monoclonal immunoglobulins. Targeted analyses have identified specific genetic abnormalities in MGUS, including IGH translocations, RB1 deletions, 1q gains, hyperdiploidy, and RAS gene mutations [125]. Somatic mutations in genes regulating cell growth, survival, and differentiation play a critical role in the pathogenesis of MGUS. In approximately half of the MGUS cases, hyperdiploidy is the founding genetic alteration. Recently, using an ingenious clonal mutation analysis approach, it was demonstrated that the first hyperdiploidy event may occur as early as the age of 20 years. As individuals age, the accumulation of age related as well as plasma cell-specific mutations and the decline in immune surveillance allow the abnormal plasma cell clones to expand, leading to the detection of M protein in the blood [90]. Age-related changes in the immune system, including immunosenescence and chronic inflammation, may create a microenvironment conducive to the emergence and persistence of these clonal plasma cells [127–130].

### Clinical implications of MGUS in the context of aging and pathogenesis of age-related diseases

While MGUS is generally asymptomatic, its presence has important clinical implications, particularly in the context of aging [87, 90]. MGUS is considered a precursor condition to more serious plasma cell disorders, such as multiple multiple myeloma [90, 131]. The gross annual risk of progression from MGUS to multiple myeloma or related malignancies is approximately 1%, necessitating regular monitoring of individuals diagnosed with MGUS [90, 128, 131]. Patients with a “high-risk” MGUS may present with an M protein > 15 g/L, and/or abnormal serum free light chain ratio and/or a non IgG M protein. If all three clinical features are present, a more advanced MGUS situation is detected and the 20-year chance for myeloma progression is 58%, significantly more than the canonical overall 1% per year value [132]. Progressive or evolving MGUS may also be recharacterized as “early myeloma” as frequently myeloma-specific genetic events (such as DNA damage- or APOBEC-related mutations) could be identified in the isolated plasma cells.

Although CHIP and MGUS share comparable annual progression rates, MGUS involves the expansion of lineage-committed cells, whereas CHIP affects hematopoietic stem cells or less mature progenitor cells. Consequently, CHIP serves as a precursor state for a wider variety of hematologic neoplasms [80]. Early detection of progression can enable timely intervention and potentially improve clinical outcomes. In addition to its potential progression to malignancy, MGUS is associated with an increased risk of other health complications, including neuropathy [90, 133], nephropathy [134–138], skin changes [139–142], and occasionally even keratopathy [143–146]. It has also been shown that the thrombosis risk is increased in MGUS, but the risk is not related to the M protein level [68]. The exact mechanisms linking MGUS to these conditions are not fully understood but may involve the effects of the monoclonal protein on various tissues and organs, as well as the underlying genetic and immune changes associated with clonal plasma cell expansion [90]. Taken together, MGUS represents a significant example of how genomic instability and clonal expansion manifest in the aging hematopoietic system. Thus, MGUS may serve as a valuable biomarker for aging and age-related diseases. Its detection can also provide insights into an individual’s risk profile for developing hematologic malignancies and other complications.

## T-cell clones of uncertain significance (TCUS)

T-cell clones of uncertain significance (TCUS) represent the clonal expansion of T-cell large granular lymphocytes (T-LGL) and are considered a likely precursor state to T-cell large granular lymphocyte leukemia (LGLL) [147]. TCUS does not present symptoms typical of lymphoproliferative disease. Evaluation for TCUS is indicated when there is a persistent presence of at least 0.5 × 10^9^/L of abnormal T lymphocytes, identified by flow cytometry as CD3 + CD8 + or CD3 + CD4 + with associated cytotoxic markers, for over 6 months, provided that other conditions, particularly viral infections, have been excluded [148]. Detection methods for TCUS include next-generation sequencing (NGS) and flow cytometry. Evidence suggests that the initial expansion of these T-cell clones is antigen-driven [149]. Genomic profiling may reveal mutations in STAT3 and STAT5B, which are predictive of malignant transformation [148]. Currently, the therapeutic approach for TCUS is a “watch and wait” strategy, similar to other premalignant conditions, as there is no effective treatment available for the condition at this time.

## Hematologic premalignant conditions as biomarkers of aging

Hematologic premalignant conditions are emerging as valuable biomarkers of aging due to their strong association with genomic instability. All these clonal conditions reflect underlying genetic alterations that accumulate with age, providing a measurable indication of genomic instability within the hematopoietic system [150, 151]. Importantly, in a recent study of 777 patients with a median age of 91 years (range 81–104), CHIP and MGUS prevalence were 17.5% and 9.5%, respectively, and the association between the two did not reach statistical significance (*p* = 0.09) [150, 152]. In another small-scale study, investigating bone marrow samples from 37 patients diagnosed with MGUS, a positive association between CHIP and MGUS was reported [153]. In this study, initial CHIP was frequent (27%) in MGUS patients, but this has to be confirmed in future larger-scale studies. The findings of the aforementioned study highlight potentially distinct pathways for CHIP and MGUS, while suggesting they share similar underlying mechanisms related to the aging process [150, 152]. The observed limited statistical association between CHIP and MGUS can be explained by the stochastic nature of mutations and clonal expansion. Since somatic mutations arise randomly, the occurrence and expansion of clonal populations in different cell types, such as hematopoietic stem cells in CHIP and plasma cells in MGUS, are not expected to have a direct one-to-one relationship. These clonal expansions are influenced by different biological processes and selective pressures within each cell population, leading to variability in their presence and extent. Therefore, while both conditions are indicative of age-related genomic instability, their independent occurrence reflects the complexity of clonal dynamics across different hematopoietic lineages. The detection of CHIP and MGUS in individuals can thus serve as a proxy for the broader genomic instability occurring throughout the body, highlighting their utility in aging research and underscoring the usefulness of incorporating both biomarkers into aging clocks to provide a more comprehensive assessment of biological aging.

In the realm of aging biomarkers, CHIP and MGUS offer unique insights compared to other widely studied measures such as epigenetic clocks. Epigenetic clocks estimate biological age based on specific patterns of DNA methylation, providing a quantifiable measure of age-related epigenetic changes. While epigenetic clocks are powerful tools for assessing biological age and predicting lifespan, CHIP and MGUS add an additional layer of information by directly indicating somatic mutations and clonal expansions. Unlike epigenetic changes, which are potentially reversible and influenced by environmental factors, the mutations leading to CHIP and MGUS are permanent and accumulate over time. This makes CHIP and MGUS particularly robust markers of cumulative genomic damage and clonal dynamics, complementing the temporal insights provided by epigenetic clocks. The integration of CHIP and MGUS with existing biomarkers in aging studies holds significant potential for enhancing our understanding of the aging process and improving risk stratification for age-related diseases [50, 64, 66, 67, 81, 98, 154]. By combining the mutation-based insights from CHIP and MGUS with the information from epigenetic, proteomic, metabolomic, and/or lipidomic clocks, it may be possible to achieve a more comprehensive assessment of the biological age of an individual.

Furthermore, the inclusion of CHIP and MGUS in longitudinal aging studies can provide valuable data on the progression of genomic instability and its impact on health over time. This can facilitate the identification of early biomarkers of disease and the development of preventative measures. As research advances, the combined use of CHIP, MGUS, and other biomarkers will likely lead to a deeper understanding of the mechanisms driving aging and the discovery of novel therapeutic targets to promote healthy aging and longevity. The ability to detect and analyze these clonal events provides a valuable window into the dynamic processes underlying aging at the molecular and cellular levels.

One promising area of investigation is the development of anti-aging therapeutic interventions based on modulation of inflammation, which plays a central role in the progression of CHIP-related diseases [49, 155–158]. Anti-inflammatory therapies, such as IL-1 inhibitors like canakinumab, have demonstrated efficacy in reducing cardiovascular events in individuals with elevated inflammation driven by clonal hematopoiesis. In addition to inflammation modulation, targeted therapies that address the specific genetic mutations commonly associated with CHIP, including DNMT3A, TET2, and ASXL1, are being explored. These interventions aim to limit clonal expansion and its downstream effects. Cardiovascular risk reduction strategies, such as lipid-lowering therapies (e.g., statins) and antithrombotic agents, are also critical in addressing the heightened risk of thrombotic events seen in CHIP carriers. Together, these approaches hold promise for reducing both the hematological and cardiovascular risks associated with clonal expansions in aging populations.

## Conclusions

In summary, premalignant hematologic conditions are powerful biomarkers that offer unique and complementary insights into genomic instability and aging. Their incorporation into aging research, alongside other biomarkers such as epigenetic clocks, can enhance the precision and predictive power of biological age assessments. By providing a more comprehensive view of the aging process, CHIP and MGUS contribute to a deeper understanding of how genomic instability drives aging and age-related pathologies [159–169]. This knowledge can lead to more effective strategies for managing and mitigating the impacts of aging on human health. Ultimately, leveraging these biomarkers can improve early detection, risk stratification, and personalized interventions, promoting healthier aging and better quality of life for older adults.
